# *TSC2* nonsense mutation in angiomyolipoma with epithelial cysts: a case report and literature review

**DOI:** 10.3389/fonc.2024.1274953

**Published:** 2024-03-25

**Authors:** Hong Song, Guoliang Mao, Nanlin Jiao, Jiajia Li, Wanwan Gao, Yinhua Liu, Linming Lu

**Affiliations:** ^1^ Department of Pathology, Wannan Medical College First Affiliated Hospital, Yijishan Hospital, Wuhu, Anhui, China; ^2^ Department of Pathology, Wannan Medical College, Wuhu, Anhui, China

**Keywords:** case report, angiomyolipoma, AMLEC, TSC2, Pi3k-akt

## Abstract

**Background:**

Angiomyolipoma with epithelial cysts (AMLEC) is an extremely rare subtype of kidney angiomyolipoma that contains epithelial-lined cysts. The most distinctive immunohistochemical feature of AMLEC is its immunoreactivity with melanocytic markers. AMLEC also has a distinct histological structure, which aids in its pathological diagnosis. To date 27 cases of AMLEC have been reported in 11 case series. However, the molecular biology underlying the pathogenesis of AMLEC remains unexplored.

**Case report:**

A 30-year-old female was diagnosed with AMLEC and underwent partial nephrectomy. Histologically, the cross-section of cystic tissue revealed a multilocular appearance, with some cysts containing thrombus-like material, and the wall thickness was approximately 0.2 ~ 0.3 cm. Additionally, the compact subepithelial cellular stroma showed strong and diffuse nuclear labeling for estrogen receptor, progesterone receptor, and CD10, as well as HMB45 and Melan A, which are markers of melanocytic differentiation. Furthermore, using a DNA targeted sequencing panel with next-generation sequencing, we identified a nonsense mutation in TSC Complex Subunit 2 (*TSC2)* gene, resulting in the formation of a premature termination codon. Moreover, the mutated genes found to be enriched in the PI3K-AKT pathway. The patient in this case had a favorable postoperative follow-up at 3 months.

**Conclusion:**

To the best of our knowledge, this study represents the first analysis of genotype mutations in AMLEC, providing valuable insights for future clinical practice. These findings have significant potential in guiding the understanding and management of AMLEC, paving the way for further research and advancements in the field.

## 1 Introduction

Renal angiomyolipoma (AML) is one of the most common benign neoplasms, typically composed of mature adipose tissue, blood vessels, and smooth muscle. In clinical practice, AMLs can be easily identified through abdominal imaging (1). However, there are same AMLs that have minimal fat, known as “fat-poor AMLs”. Among them, angiomyolipoma with epithelial cysts (AMLEC) is an exceptionally rare subtype of fat-poor AML. Histologically, AMLEC is characterized by the presence of epithelial-lined cysts and a thick exterior wall consisting of poorly formed fascicles of smooth muscle and dysplastic blood vessels with thick walls (2). Immunohistochemical imaging of AMLECs typically exhibits distinct features and shows positive reactivity for melanocytic markers, estrogen and progesterone receptors, such as HMB45, Melan A, ER, PR, CD10 and WT-1. AMLECs may manifest either incidentally or with associated symptoms commonly including flank pain and hematuria. While the majority of AMLEC lesions are benign, there is a rare possibility of cystic lesions being malignant, necessitating surgical excision.

To best of our knowledge, there is limited information about the imaging findings of AMLEC available. To date, only a total of 27 cases have been reported in 11 published case reports (3–14). Furthermore, there are only two reports that present the immunohistochemical and imaging characteristics of a combination of AMLEC and EAML another variant of AML (6, 15). Notably, there have been no reports of progression or metastasis during the indolent clinical course of AMLEC. However, the molecular biology underlying the pathogenesis of AMLEC remains unaddressed in the literature.

*TSC2* (TSC Complex Subunit 2) is a crucial tumor suppressor gene responsible for encoding the growth inhibitory protein tuberin. Tuberin interacts with hamartin to form the TSC protein complex, which plays a vital role in regulating cell growth. Specifically, this TSC protein complex exerts a negative control on the signaling pathway of mammalian target of rapamycin complex 1 (mTORC1), a key regulator of anabolic cell growth. It is worth noting that mutations in this gene have been associated with tuberous sclerosis and lymphangioleiomyomatosis (16). To date, there have been no reports have linked *TSC2* mutations to AMLEC.

So, in this study, we not only reported the histological and immunohistochemical features of AMLEC, but also identified a nonsense mutation in TSC2 using a DNA targeted sequencing panel with next-generation sequencin (NGS). Furthermore, we observed multiple mutation types in a total of 20 genes, and KEGG analysis indicated enrichment of the PI3K-AKT pathway. Notably, the *TSC2* nonsense mutation discovered in this study has the potential to be a clinically significant variant that could confer sensitivity to everolimus.

## 2 Case presentation

The patient is a 30-year-old female who presented with discomfort and pain in the right flank area. A lump was discovered during physical examination specifically located between the liver and right kidney in the abdomen. The patient had no personal or family history of chronic diseases such as hypertension, diabetes, or coronary heart disease. Additionally, there was no history of renal masses, renal cell carcinoma, angiomyolipomas, tuberous sclerosis, or any similar conditions in the patient or her family. The physical examination revealed a negative Murphy’s sign for the gallbladder, and the spleen was not palpable. There were no apparent signs of edema in both lower limbs, and the nervous system appeared normal.

**Figure 1:**
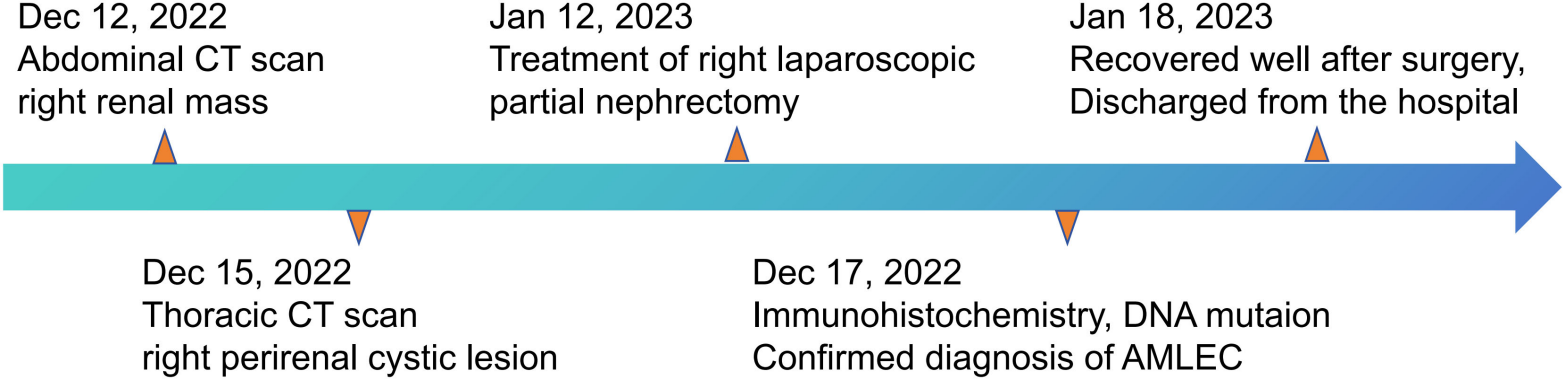
The clinical course schedule of this patient showing the treatment route received by the patient.

The abdominal computed tomography (CT) scan, which included both plain and contrast enhancement, identified a cystic density lesion measuring 63 mm × 39 mm in the right kidney, which was classified as Bosniak III type. On January 12th, 2023, the patient underwent a right partial nephrectomy. The treatment route followed by the patient is summarized in **Figure 1**.

**Figure 2:**
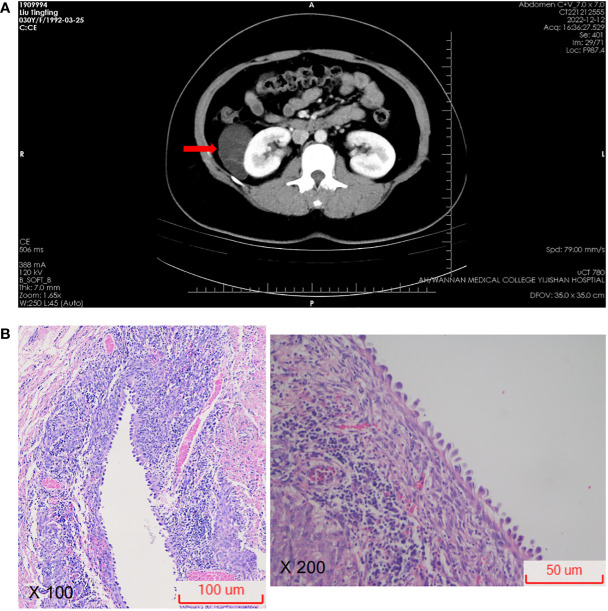
Radiological and microscopic findings. **(A)** On computed tomography, a cystic density lesion measuring 63 mm × 39 mm was found at the upper part of the right kidney (red arrow). **(B)** Photomicrograph showing epithelial cysts lined by cuboidal to hobnailed cells (H&E100X, H&E200X).

**Figure 3:**
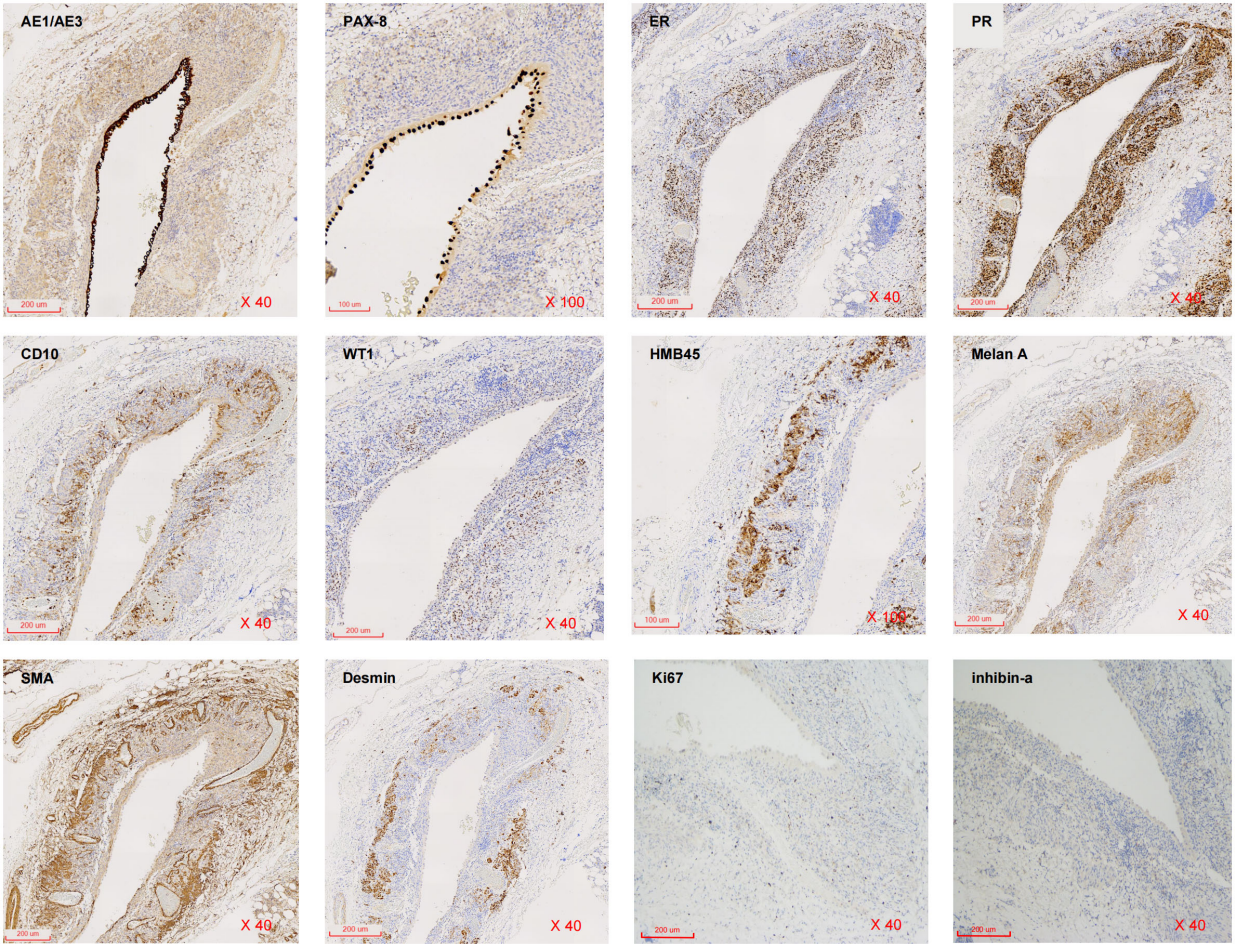
Immunohistochemical findings. Immunohistochemistry for AE1/AE3, PAX-8, ER, PR, CD10, WT1, HMB45, Melan A, SMA, Desmin, Ki67 and inhibin-a expression in AMLEC lesions (from right to light, from top to below) (H&E40X).

The border of the cyst was still clear, and it protruded outward locally (**Figure 2A**). Subsequently, the patient was transferred to the urology department for further diagnosis and treatment. Upon admission, the patient was diagnosed with a “right renal occupying lesion”. A gray-yellow-gray-white cystic tissue measuring approximately 6.0 cm × 4.5 cm × 2.8 cm was submitted for histopathological and Immunohistochemical examination. Histopathologically, the sliced specimen revealed a multilocular cyst with some cysts containing thrombus-like material. The cyst wall measured approximately 0.2cm-0.3cm in thickness (**Figure 2B**). Immunohistochemically, the epithelial cells lining the cyst wall showed diffuse expressed of AE1/AE3 and PAX-8 (**Figure 3**), suggesting that the cysts may have resulted from cystic dilatation of collecting ducts that invaginated into the tumor. The compact subepithelial cellular stroma exhibited strong and diffuse nuclear labeling for estrogen receptor (ER), progesterone receptor (PR), and cytoplasmic labeling for CD10. However, labeling for ER and PR was patchy in the smooth muscle-like cells. Additionally, HMB45 and Melan A (**Figure 3**), markers of melanocytic differentiation, were most intense and concentrated in the subepithelial cells, while HMB45 showed patchy labeling in the exterior muscle-predominant AML component. Meanwhile, SMA and Desmin were specifically labeled in smooth muscle-like cells. S-100 protein did not label any of the three components of the tumor, except for patchy labeling of subepithelial cells. The tumor exhibited a low proliferative index, with Ki67 (**Figure 3**) labeling less than 1% of neoplastic cells. Furthermore, CA IX, CD117 (c-kit), CD34 (endothelial markers), and inhibin-a did not label any of the three components of the tumor. Based on all these findings, we diagnosed the predominantly cystic tumor as AMLEC, accompanied by vascular smooth muscle lipoma and an epidermal cyst.

Simultaneously, the patient’s tumor biospecimen and circulating tumor DNA in plasma (ctDNA) nearby the tumor were sent to Geneseeq Technology Inc. for NGS analysis. The NGS test covered approximately 1.53 Mb base pairs, including exons of 437 genes, fusion-related introns, variable splicing regions, and specific microsatellite (MS) loci. The test results provided information on point mutations, small fragment insertion and deletion mutations, gene fusion and copy number variations, microsatellite (MS) analysis results, and tumor mutation burden (TMB) information within the coverage range.

In the AMLEC tumor sample, a low TMB of 1.0 mutation per megabase (Mb) was observed, and no germline mutations were found when compared with ctDNA. Mutations were identified in a total of 20 genes, as detailed in **Table 1**, with additional information available in **Supplementary Table S1**. Notably, no mutations were identified in four mismatch repair-related genes: MLH1, MSH2, MSH6, and PMS2. Additionally, the analysis revealed 10 polymorphic mutations associated with drug metabolism enzymes. Specifically, a nonsense mutation of c.2157T>A was detected in the 20th exon of the TSC2 gene. This mutation resulted in the substitution of the 719th amino acid from tyrosine to a stop codon, leading to premature termination of protein synthesis and the production of a truncated protein. The presence of mutations in these 20 genes suggests enrichment in pathways related to platinum drug resistance, PI3K-Akt signaling pathway, and nucleotide excision repair. These findings provide potential implications for targeted therapies and insights into the molecular mechanisms underlying the development and progression of AMLEC (**Supplementary Figure S1**).

**Table 1:** Gene list of 20 genes with mutations in our AMLEC case.

id	Type	Gene	Gene.ID	AAChange	Ref	Alt	Hom.Het	ExonicFunc
FC232L0241	SNP	BCL2L11	.	.	.	.	het	.
FC232L0241	SNP	DPYD	DPYD : NM_000110.4:exon13	c.1627A>G(p.I543V)	T	C	hom	missense_variant
FC232L0241	SNP	ERCC1	ERCC1:NM_001983.4:exon4	c.354T>C(p.N118=)	A	G	hom	synonymous_variant
FC232L0241	SNP	ERCC2	ERCC2:NM_000400.3:exon23	c.2251A>C(p.K751Q)	T	G	het	missense_variant
FC232L0241	SNP	GSTP1	GSTP1:NM_000852.4:exon5	c.313A>G(p.I105V)	A	G	het	missense_variant
FC232L0241	SNP	NQO1	NQO1:NM_000903.3:exon6	c.559C>T(p.P187S)	G	A	het	missense_variant
FC232L0241	SNP	UGT1A1	UGT1A1:NM_000463.3:exon1	c.211G>A(p.G71R)	G	A	het	missense_variant
FC232L0241	SNP	UGT1A1	UGT1A1:NM_000463.3:2	.	–	AT	het	upstream_gene_variant
FC232L0241	SNP	XRCC1	XRCC1:NM_006297.2:exon10	c.1196A>G(p.Q399R)	T	C	hom	missense_variant
FC232L0241	SNP	GSTM1	.	.	.	.	hom	.
FC232L0241	Germline	ERCC5	ERCC5:NM_000123.3:exon9	c.2108A>G(p.D703G)	A	G	het	missense_variant
FC232L0241	Germline	POLE	POLE : NM_006231.4:exon35	c.4541T>C(p.V1514A)	A	G	het	missense_variant
FC232L0241	Germline	ALK	ALK : NM_004304.5:exon26	c.3837G>T(p.R1279S)	C	A	het	missense_variant
FC232L0241	Germline	BRCA1	BRCA1:NM_007294.4:exon11	c.2726A>T(p.N909I)	T	A	het	missense_variant
FC232L0241	Mutant	TSC2	TSC2:NM_000548.5:exon20	c.2157T>A(p.Y719*)	T	A	het	stop_gained
FC232L0241	MS	.	.	MSS	.	.	.	.
FC232L0241	TMB	.	.	.	.	.	.	.
FC232L0241	check.germ	CEBPA	CEBPA:NM_004364.4:exon1	c.365G>A(p.G122E)	C	T	het	missense_variant
FC232L0241	check.germ	KDM5A	KDM5A:NM_001042603.3:exon4	c.434G>A(p.R145H)	C	T	het	missense_variant
FC232L0241	check.germ	SDC4	SDC4:NM_002999.4:exon5	c.511C>T(p.R171C)	G	A	het	missense_variant
FC232L0241	check.germ	RUNX1	RUNX1:NM_001001890.3:exon6	c.924G>T(p.Q308H)	C	A	het	missense_variant
FC232L0241	check.germ	ERBB3	ERBB3:NM_001982.3:exon9	c.1034C>T(p.S345L)	C	T	het	missense_variant
FC232L0241	check.germ	TTF1	TTF1:NM_007344.4:exon10	c.2398G>A(p.V800I)	C	T	het	missense_variant

## 3 Discussion

AML is a well-known and frequently encountered neoplasm characterized by a triphasic composition of adipose tissue, thick-walled vessels, and epithelioid smooth muscular. However, there are instances where AMLs exhibit insufficient amounts of fat, posing challenges in their detection using unenhanced CT scans. In such cases, misdiagnosed as renal cell carcinoma may occur. AMLEC, a rare variant of AML, presents a particular diagnostic challenge due to its minimal fat content and the presence of epithelial cysts. Unlike typical AMLs, AMLECs exhibit both solid and cystic areas, accompanied by an epithelial component. Therefore, when evaluating cystic renal neoplasms in adults, it is crucial to consider AMLEC as a potential differential diagnosis. It is worth noting that AMLECs are less frequently associated with tuberous sclerosis compared to conventional AMLs (5, 17).

Currently, there are no specific tumor markers or imaging characteristics that can definitively diagnose AMLEC prior to surgery. The accurate diagnosis of AMLEC relies solely on histopathological and immunohistochemical findings. When considering the differential diagnosis, AMLEC stands out as distinct from most adult cystic renal tumors. It is crucial to rule out other conditions such as mixed epithelial and stromal tumor (MEST), cystic nephroma, multilocular cystic renal cell carcinomas, and cystic partially differentiated nephroblastoma. It is worth noting that AMLEC and MEST share some morphological similarities, including cysts lined by cuboidal or hobnail epithelia and smooth muscle walls. Additionally, both AMLEC and MEST exhibit immunoreactivity for ER, PR, and CD10 in their stromal cells. However, the presence of dysplastic features in the vessels of AMLEC, which are absent in MEST, can serve as an important distinguishing factor. Furthermore, the presence of melanocytic markers HMB45 and Melan A is a significant positive indicator for AMLEC, while MEST does not exhibit immunoreactivity for these markers.

The clinical features of AMLEC do not appear to differ significantly from classic AMLs. AMLs are generally considered to be benign and exhibit an indolent clinical course. Based on reported cases of AMLECs, all patients underwent either partial nephrectomy (including our case) or radical nephrectomy. Only one case resulted in the patient’s death, which was unrelated to AMLEC but due to another underlying disease. Additionally, there have been two reported cases of a combination of AMLEC and EAML, which were also confirmed based on immunohistochemical and imaging studies (6, 15). It is crucial to establish appropriate timing and frequency for follow-up examinations, but chemotherapy or radiation therapy is unnecessary for AMLEC (15).

We detected a nonsense mutation of p.Y719* in the 20th exon of the *TSC2* gene, which caused a stop codon and the production of a truncated protein. It is reported that AML with multiple lesions is closely associated with tuberous sclerosis complex (TSC), which shares the same name as the gene responsible for its development. However, the sporadic solo lesion AMLEC has not been reported to have any known relationship with mutations in the *TSC2* genes. The presence of this truncated protein could potentially lead to the inactivation of the TSC2 protein. Consequently, it may result in a reduction in the hydrolysis of downstream RHEB molecules in the mTOR pathway, leading to an accumulation of RHEB GTP levels. This, in return, activates the mTOR pathway, ultimately contributing to tumor development by enhancing the cell’s anti-apoptotic ability. Moreover, this mutation may also increase the sensitivity to mTOR inhibitors (18). Similarly, mutations in the *TSC2* gene can lead to the development of TSC by causing hyperactivation of the mTOR pathway. In recent years, the mTOR inhibitor everolimus has been approved as an adjunctive therapy for TSC-associated partial seizures (19). It is reasonable to propose that everolimus, as mTOR inhibitor, holds promising clinical significance as an adjunct therapy for AMLEC.

## 4 Conclusion

In this study, we present a rare case of AMLEC in a 30-year-old female patient. AMLEC is an extremely rare benign kidney tumor, and the main treatment options are partial nephrectomy (as in our case) or radical nephrectomy. AMLECs typically appear as radiographically and grossly cystic renal lesions, emphasizing the importance of distinguishing them from other cystic renal neoplasms. A notable aspect of this case is the presence of three distinct morphological patterns and three different immune phenotypes. While AMLEC may resemble MEST, its most distinctive feature is its immunoreactivity not only to ER, PR, and CD10, but also to melanocytic markers such as Melan-A and HMB45. Understanding the characteristics of this disease will help differentiate predominantly cystic renal tumors. Currently, there is no literature or clinical reports specifically addressing the relationship between TSC2 nonsense mutation and AMLEC, apart from this case. However, given the potential implications of TSC2 mutations in the pathogenesis of AMLEC, further investigation is necessary to assess its role as a potential target for diagnosis and treatment in future clinical research.

There were some limitations in our study. Firstly, we only had one AMLEC case, which is a small sample size for AMLEC treatment. In the future, we plan to increase the sample size to obtain more generalizable ultrasound features that can provide references for the diagnosis and treatment of AMLEC. Secondly, the TSC2 nonsense mutation identified in this case may be a specific mutation, and its clinical significance as an adjunct therapy for AMLEC needs further exploration. Additionally, more cases are needed to verify this mutation and its therapeutic effect.

## Data availability statement

The datasets presented in this study can be found in online repositories. The names of the repository/repositories and accession number(s) can be found below: https://ngdc.cncb.ac.cn/search/?dbId=hra&q=PRJCA018538, PRJCA018538.

## Ethics statement

The studies involving humans were approved by Medical Ethics Committee of Wannan Medical College. The studies were conducted in accordance with the local legislation and institutional requirements. The participants provided their written informed consent to participate in this study. Written informed consent was obtained from the individual(s) for the publication of any potentially identifiable images or data included in this article.

## Author contributions

LL: Data curation, Funding acquisition, Resources, Writing – original draft, Writing – review & editing. HS: Data curation, Investigation, Writing – original draft, Writing – review & editing. GM: Data curation, Formal analysis, Methodology, Writing – original draft, Writing – review & editing. NJ: Data curation, Investigation, Formal analysis, Writing – original draft. JL: Data curation, Supervision, Conceptualization, Project administration, Writing – original draft. WG: Data curation, Investigation, Supervision, Validation, Writing – original draft. YL: Formal analysis, Validation, Writing – review & editing.

## Supplementary material

Supplementary Figure 1 KEGG pathways enriched by 20 genes with mutations as shown in **Table 1**.
