# Supplementary figures and images for: TSC2 nonsense mutation in angiomyolipoma with epithelial cysts: a case report and literature review
Source: Front Oncol. 2024 Mar 25;14:1274953. doi: 10.3389/fonc.2024.1274953 (PMC10999537; doi:10.3389/fonc.2024.1274953)

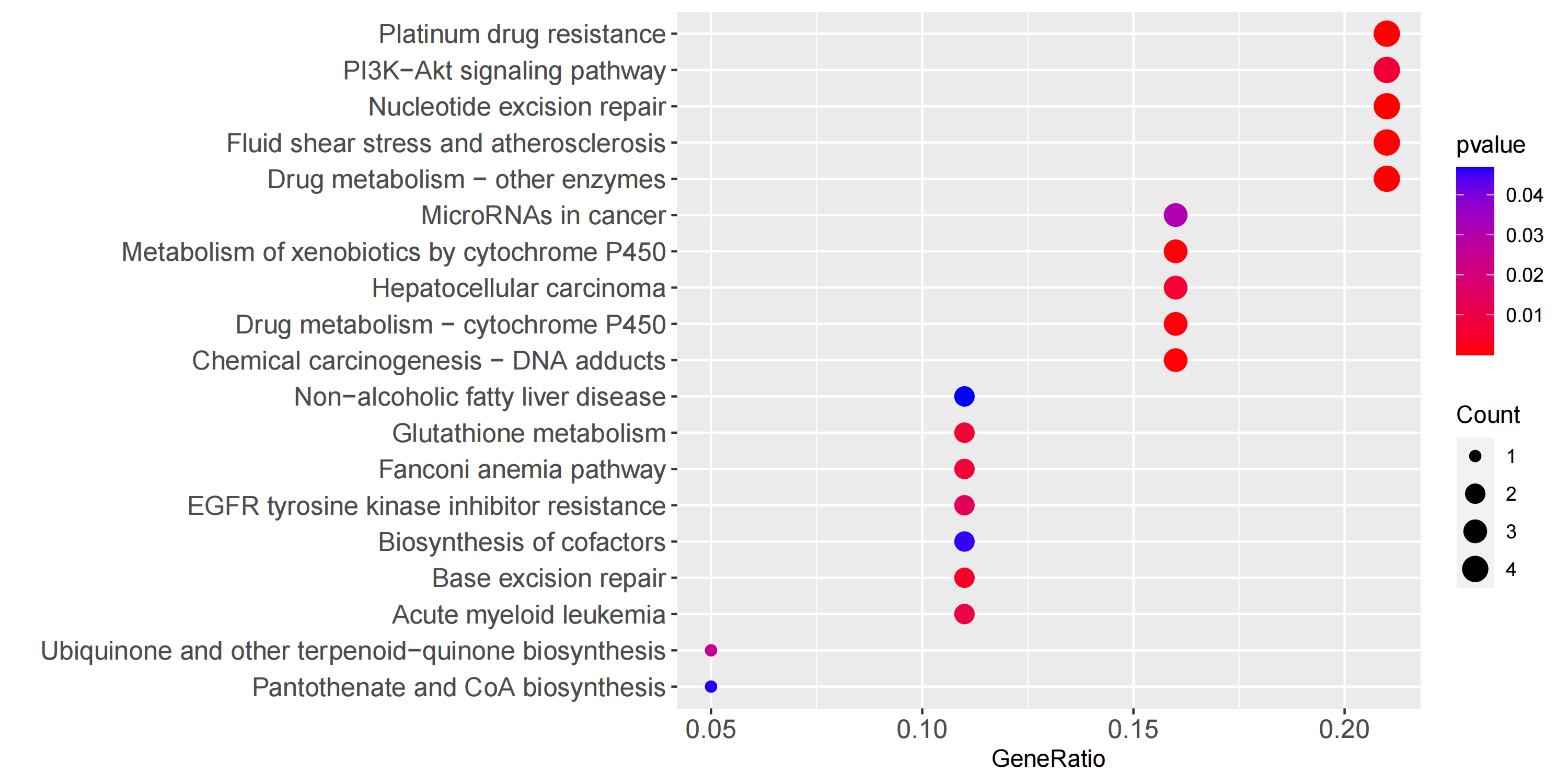

Supplement: Supplementary Figure 1 — KEGG pathways enriched by 20 genes with mutations as shown in Table 1 . [file Image_1.tif]
